# Longitudinal serum metabolomics evaluation of trastuzumab and everolimus combination as pre-operative treatment for HER-2 positive breast cancer patients

**DOI:** 10.18632/oncotarget.18784

**Published:** 2017-06-28

**Authors:** Elodie Jobard, Olivier Trédan, Thomas Bachelot, Arnaud M. Vigneron, Céline Mahier Aït-Oukhatar, Monica Arnedos, Maria Rios, Jacques Bonneterre, Véronique Diéras, Marta Jimenez, Jean-Louis Merlin, Mario Campone, Bénédicte Elena-Herrmann

**Affiliations:** ^1^ Université de Lyon, Institut des Sciences Analytiques, UMR 5280, CNRS, Université Lyon 1, ENS de Lyon, Villeurbanne, France; ^2^ Université de Lyon, Centre Léon Bérard, Département d’oncologie médicale, Lyon, France; ^3^ Université de Lyon, Centre de Cancérologie de Lyon, UMR Inserm 1052 CNRS 5286, Centre Léon Bérard, Lyon, France; ^4^ R&D Unicancer, UNICANCER, Paris, France; ^5^ Department of Medicine, Gustave Roussy, Villejuif, France; ^6^ Department of Medical Oncology, Centre Alexis Vautrin, Vandoeuvre-les-Nancy, France; ^7^ Department of Medical Oncology, Centre Oscar Lambret, Lille, France; ^8^ Department of Medical Oncology, Institut Curie, Paris, France; ^9^ CNRS UMR7039 CRAN, Université de Lorraine, Vandoeuvre-les-Nancy, France; ^10^ Department of Biopathology Unit, Institut de Cancérologie de Lorraine, Vandoeuvre-Les-Nancy, France; ^11^ Institut de Cancérologie de l’Ouest, Centre René Gauducheau, Saint-Herblain, France

**Keywords:** HER-2 positive breast cancer, metabolomics, nuclear magnetic resonance, targeted therapies, mTOR inhibitor

## Abstract

The mammalian target of rapamycin complex 1 (mTORC1) is an attractive target for HER-2 positive breast cancer therapy because of its key role in protein translation regulation, cell growth and metabolism. We present here a metabolomic investigation exploring the impact of mTOR inhibition on serum metabolic profiles from patients with non-metastatic breast cancer overexpressing HER-2.

Baseline, treatment-related and post-treatment serum samples were analyzed for 79 patients participating in the French clinical trial RADHER, in which randomized patients with HER-2 positive breast cancer received either trastuzumab alone (arm T) or a trastuzumab and everolimus combination (arm T+E). Longitudinal series of NMR serum metabolic profiles were exploited to investigate treatment effects on the patients metabolism over time, in both group.

Trastuzumab and everolimus combination induces faster changes in patients metabolism than trastuzumab alone, visible after only one week of treatment as well as a residual effect detectable up to three weeks after ending the treatment. These metabolic fingerprints highlight the involvement of several metabolic pathways reflecting a systemic effect, particularly on the liver and visceral fat. Comparison of serum metabolic profiles between the two arms shows that everolimus, an mTORC1 inhibitor, is responsible for host metabolism modifications observed in arm T+E.

In HER-2 positive breast cancer, our metabolomic approach confirms a fast and persistent host metabolism modification caused by mTOR inhibition.

## INTRODUCTION

For about 20-30% of patients with breast cancer, cancer cells overexpress a growth-promoting HER/neu protein on their surface. This cancer, known as HER-2 positive breast cancer (HER-2+) is characterized by an aggressive disease progression and poor prognosis [1]. Many advances in recent years, such as the use of targeted therapies, have enabled improvement in the management of HER-2+ breast cancer patients. Administration of trastuzumab, a recombinant monoclonal antibody against HER-2, proved as a truly appropriate treatment for patients with HER-2+ breast cancer and has improved their prognosis. Numerous clinical trials have positively evaluated the activity of trastuzumab, in combination with various chemotherapy agents, in terms of response rate, overall survival and risk of relapse [2, 3]. Moreover, in recent years, several targeted therapies for HER-2+ tumors including pertuzumab, lapatinib and trastuzumab emtansine have been approved for treatment of metastatic HER-2+ breast cancer. Others agents targeting several molecular pathways implicated in trastuzumab resistance have also shown encouraging results in advanced HER-2+ disease [1], notably the mammalian target of rapamycin (mTOR) inhibitor everolimus [4–6].

mTOR is an attractive target for cancer therapeutic intervention. The mTOR protein is a serine/threonine kinase that plays a crucial role in regulating various signaling pathways (PI3K/Akt, TSC, Ras, protein and lipid biosynthesis), and as such serves as a central regulator of cell growth, proliferation, survival and metabolism. Deregulation of the mTOR-signaling pathway (*PIK3CA* amplification/mutation, *PTEN* loss of function, Akt overexpression, and S6K1, 4EBP1 and eIF4E overexpression) is associated with several human disorders such as diabetes, obesity and cancer. Upstream regulators and downstream effectors of the mTOR pathway have been widely described in recent reviews [7–9]. In this context, the RADHER trial was set up to evaluate the effectiveness of combining trastuzumab and everolimus in pre-operative treatment of early breast cancer (EBC), as compared with trastuzumab treatment alone.

Metabolomics investigations are increasingly used in breast cancer research. Initial studies primarily intended to identify biomarkers discriminating benign vs. malignant tissue samples [10, 11] and subtypes of breast cancer [12, 13]. More recently, a growing number of studies on human biological fluids (blood and urine) have aimed at highlighting biomarkers distinguishing early breast cancer and relapses [14–16] or subclasses linked to cancer treatment response [17–20]. Miolo and coworkers [19] have highlighted predictive biomarkers associated with response to neoadjuvant therapy (trastuzumab-paclitaxel) in HER-2+ breast cancer.

In this work, we present a metabolomic investigation exploring the impact of mTOR inhibition on the serum metabolic profiles of patients with EBC overexpressing HER-2. We detail the metabolic signatures associated with response to trastuzumab, or a combination of trastuzumab and everolimus.

## RESULTS

### Patients characteristics

To investigate the metabolic changes associated with targeted therapies, 79 patients with HER-2+ EBC from the RADHER clinical trial, treated with either trastuzumab alone (arm T: 40 patients) or a combination of trastuzumab and everolimus (arm T+E: 39 patients) were included in our metabolomics analysis. Principal characteristics of these patients are summarized in Table 1. Biological and clinico-pathological data evaluation reveals no significant differences between arms T and T+E, excluding bias related to patients’ selection. According to the Sataloff classification, 48.7% of patients who were administered the everolimus and trastuzumab combination display partial or complete response to treatment while only 42.5% show similar response within arm T.

**Table 1:** Clinicopathological characteristics of the RADHER trial patients

Characteristics	Arm T	Arm T+E	*p*-value ^a^
**No. of subjects**	40 (50.6%)	39 (49.4%)	
**Age (median/SD)**	50 (±13.3)	51 (±12.2)	0.48
**No. of serum samples**			
W0	16	23	
W1	36	34	
W4	36	31	
W7	27	30	
W9	24	28	
W13	29	26	
**Menopausal status**			0.65
Pre-Menopausal	22 (55%)	19 (48.7%)	
Post-Menopausal	18 (45%)	20 (51.3%)	
**BMI (body mass index)**			1
≤ 25	24 (60%)	23 (59%)	
> 25	15 (37.5%)	16 (41%)	
Unknown	1 (2.5%)	0 (0%)	
**Hormone receptors ^b^**			0.65
HR +	18 (45%)	15 (38.5%)	
HR -	22 (55%)	24 (61.5%)	
**Size tumor residue**			0.30
≤ 2 cm	18 (45%)	24 (61.5%)	
> 2 cm	13 (32.5%)	10 (25.7%)	
Unknown	9 (22.5%)	5 (12.8%)	
**Tumor type**			0.39
Ductal	33 (82.5%)	36 (92.3%)	
Lobular	1 (2.5%)	0 (0%)	
Others	6 (15%)	3 (7.7%)	
**SBR grade ^c^**			0.05
1	0 (0%)	1 (2.6%)	
2	12 (30%)	22 (56.4%)	
3	20 (50%)	11 (28.2%)	
Unknown	8 (20%)	5 (12.8%)	
**Sataloff classification ^d^**			0.23
Complete Response	6 (15%)	3 (7.7%)	
Partial Response	11 (27.5%)	16 (41%)	
No Response	21(52.5%)	20 (51.3%)	
No information	2 (5%)	0 (0%)	
**Toxicity ^e^ at W4**			0.01
Grade 1 & 2	35 (87.5%)	30 (76.9%)	
Grade 3 & 4	0 (0%)	7 (17.9%)	
No toxicity	2 (5%)	0 (0%)	
No information	3 (7.5%)	2 (5.1%)	

^a^ *p-*value calculated using either the χ^2^ or Fisher exact tests for proportions or a Student test for median.

^b^ Hormone receptors are receptors for estrogen and progesterone; HR-: at least one of the two receptors are negative; HR+: both receptors are positive.

^c^ SBR (Scarff Bloom and Richardson) grade consists in three grades obtained by addition of three criteria: architecture, shape and size of the nuclei and number of dividing cells.

^d^ Pathological response rate is centrally evaluated according to Sataloff classification (complete response: T-A; partial response: T-B; no response: T-C and T-D).

^e^ Toxicity data was recorded after four weeks following NCI CTC criteria. The toxicity corresponds to the maximum intensity for all types of toxicities.

### Untargeted ^1^H NMR-based metabolomics analysis

**Figure 1:**
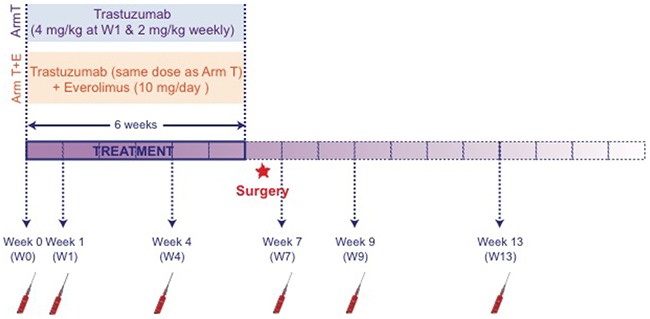
Study Design of the RADHER trial Women patients with early breast cancer (EBC) overexpressing HER-2 were randomized using a 1:1 ratio. Group T was treated with trastuzumab alone; group T+E was administered a combination of trastuzumab and everolimus. Blood samples were collected under fasting conditions, at six different times: at baseline (W0) i.e. before the first therapy cure; one (W1) and four weeks (W4) after the beginning of the treatment; two weeks (W7), four weeks (W9) and eight weeks (W13) after the end of the treatment, i.e. after the last drip of trastuzumab. NMR analysis was performed once the trial completed.

Serum samples collected for each patient at different time points before (W0), during targeted therapy (W1 and W4), and after drug intervention (W7, W9 and W13) were analyzed by ^1^H NMR spectroscopy. The global study design is summarized in Figure 1. ^1^H NMR metabolic profiles were thus obtained for a total of 341 serum samples (W0: 39, W1: 70, W4: 67, W7: 57, W9: 52 and W13: 56), corresponding to 168 samples from 40 patients in group T (W0: 16, W1: 36, W4: 36, W7: 27, W9: 24 and W13: 29) and 172 samples from 39 patients following treatment T+E (W0: 23, W1: 34, W4: 31, W7: 30, W9: 28 and W13: 26), and further evaluated using multivariate statistical analyses. Supervised analyses of the metabolic profiles carried out in relation with tumor's characteristics (tumor type, hormone receptors, size tumor residue, SBR grade, Sataloff classification and toxicity) showed an absence of significant associations (Supplementary Table 1), allowing to neglect in the following the specific impact of tumor characteristics on serum profiles.

### Metabolic profiles associated with treatments of HER-2+ EBC

**Figure 2:**
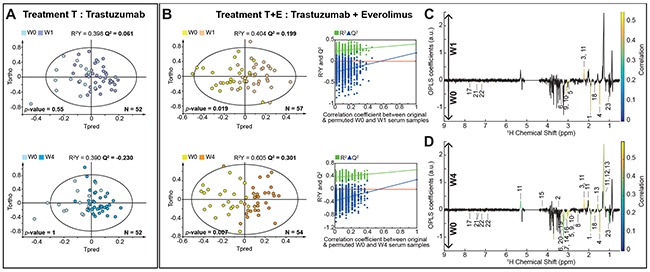
Discrimination between pre- and on-treatment serum samples **(A)** O-PLS models score plots for group T evaluating samples discrimination at W0 vs. W1 (1+1 components, R^2^X = 0.457, R^2^Y = 0.398, Q^2^ = 0.061, CV-ANOVA *p-*value = 0.55) and W0 vs. W4 (1+1 components, R^2^X = 0.487, R^2^Y = 0.39, Q^2^ = -0.230, CV-ANOVA *p*-value = 1). **(B)** O-PLS models score plots for group T+E, discriminating samples at W0 vs. W1 (1+1 components, R^2^X = 0.497, R^2^Y = 0.404, Q^2^ = 0.199, CV-ANOVA *p-*value = 0.019) and W0 vs. W4 (1+2 components, R^2^X = 0.602, R^2^Y = 0.605, Q^2^ = 0.301, CV-ANOVA *p*-value = 0.007). O-PLS model validations by re-sampling 1000 times the model under the null hypothesis for the treatment T+E. **(C) and (D)** O-PLS loadings plots represented for group T+E: W0 vs. W1 and W0 vs. W4, respectively. Statistically significant individual signals correspond to the colored spectral regions. Highlighted candidate markers are: 1) Acetate, 2) Acetoacetate, 3) Acetone, 4) Alanine, 5) Albumin Lysyl, 6) Betaine, 7) Choline, 8) Citrate, 9) Creatine, 10) Creatinine, 11) Fatty acids, 12) Fatty acids (mainly LDL), 13) Fatty acids (mainly VLDL), 14) Glucose, 15) Glycerol backbone of PGLYs and TAGs, 16) Glycerophosphocholine, 17) Histidine, 18) Lysine, 19) Methanol, 20) Myo-inositol, 21) Phenylalanine, 22) Tyrosine, 23) Valine.

To probe the specific metabolic response associated with trastuzumab alone or trastuzumab and everolimus treatments, NMR metabolic profiles were first evaluated within each arm of the study. Supervised multivariate statistics (O-PLS models) were built to compare metabolic profiles and derive robust statistical models discriminating pre- versus on-treatment metabolic serum profiles (Figure 2).

Regarding the group T, O-PLS score plots obtained from serum metabolic profiles between pre- and on-treatment samples (W0 and W1, W0 and W4) revealed no significant separation (Figure 2A). In contrast, O-PLS models discriminate for group T+E serum metabolic profiles at W0 versus W1 (R^2^X = 0.497, R^2^Y = 0.404, Q^2^ = 0.199, CV-ANOVA *p-*value = 0.019) and at W0 versus W4 (R^2^X = 0.581, R^2^Y = 0.603, Q^2^ = 0.301, CV-ANOVA *p-*value = 0.001 × 10^-5^). Statistical significance for these two models is assessed by high values of goodness-of-fit parameters R^2^ and Q^2^, CV-ANOVA *p*-values < 0.05, and model resampling under the null hypothesis (Figure 2B). Univariate analyses further identified significant changes in individual metabolite concentrations between baseline and on-treatment samples for group T+E. As illustrated in Figure 2C, administration of the everolimus and trastuzumab combination is correlated at W1 with an increase in fatty acids and acetone concentrations and a relative decrease in acetate, amino acids (alanine, histidine, lysine, phenylalanine, tyrosine and valine), albumin lysyl, betaine, creatine and creatinine (*p* < 0.05). All statistically relevant metabolites, corresponding fold-changes and *p-*values are reported in Table 2.

**Table 2:** Metabolites significantly associated with treatment T+E

ID	Name	W0 vs. W1			W0 vs. W4			W0 vs. W7			W0 vs. W9			Variation
		*p-*value^a^	*q*-value BH^b^	Fold Change	*p-*value^a^	*q*-value BH^b^	Fold Change	*p-*value^a^	*q*-value BH^b^	Fold Change	*p-*value^a^	*q*-value BH^b^	Fold Change	
**1**	Acetate	0.003	0.038	0.73	0.003	0.019	0.71	0.001	0.006	0.66				↓
**2**	Acetoacetate				0.009	0.042	0.84	0.001	0.009	0.79	0.001	0.023	0.83	↓
**3**	Acetone	0.0006	0.025	1.27	0.0001	0.002	1.44	0.0001	0.002	1.39				↑
**4**	Alanine	0.001	0.032	0.83	0.001	0.010	0.80	0.001	0.006	0.81				↓
**5**	Albumin Lysyl	0.002	0.038	0.83	0.00004	0.002	0.77	0.0006	0.006	0.81				↓
**6**	Betaine	0.004	0.038	0.82	0.0003	0.005	0.76	0.002	0.012	0.77				↓
**7**	Choline				0.007	0.040	0.88	0.003	0.016	0.86				↓
**8**	Citrate				0.002	0.015	0.80							↓
**9**	Creatine	0.001	0.032	0.83	0.00001	0.001	0.78	0.000001	0.00001	0.76				↓
**10**	Creatinine	0.00002	0.002	0.86	0.000002	0.0003	0.78	0.000001	0.00001	0.76				↓
**11**	Fatty acids	0.0006	0.025	1.27	0.010	0.046	1.23	0.001	0.009	1.32	0.003	0.041	1.12	↑
**12**	Fatty acids (mainly LDL)				0.002	0.015	1.32	0.001	0.006	1.37				↑
**13**	Fatty acids (mainly VLDL)				0.002	0.015	1.35	0.002	0.012	1.35				↑
**14**	Glucose				0.002	0.018	0.86	0.0002	0.004	0.82	0.0003	0.013	0.84	↓
**15**	Glycerol backbone of PGLYs & TAGs				0.003	0.019	1.15	0.001	0.006	1.21	0.0001	0.013	1.20	↑
**16**	Glycerophosphocholine				0.007	0.040	0.82	0.001	0.006	0.79				↓
**17**	Histidine	0.004	0.040	0.85	0.0003	0.005	0.82	0.0004	0.006	0.78				↓
**18**	Isoleucine							0.008	0.035	0.85				↓
**19**	Lysine	0.001	0.032	0.85	0.001	0.008	0.81	0.0001	0.002	0.79				↓
**20**	Mannose							0.009	0.036	1.30				↑
**21**	Methanol				0.002	0.016	0.80	0.001	0.008	0.78				↓
**22**	Myo-inositol				0.0001	0.003	0.78	0.0001	0.002	0.75				↓
**23**	NAC 1							0.002	0.011	1.10	0.0003	0.013	1.15	↑
**24**	Phenylalanine	0.0003	0.019	0.64	0.0003	0.005	0.58	0.001	0.008	0.63	0.002	0.037	0.59	↓
**25**	Proline							0.001	0.008	0.78				↓
**26**	Tyrosine	0.002	0.038	0.83	0.00001	0.001	0.74	3 × 10 ^-8^	0.00001	0.67	0.0002	0.012	0.78	↓
**27**	Valine	0.004	0.038	0.81	0.010	0.046	0.89	0.00002	0.0008	0.70	0.004	0.044	0.86	↓

^a^ *p-*value without multiple testing correction.

^b^ *q-*value BH: *p-*value after Benjamini-Hochberg false discovery rate multiple testing correction. Variation: ↑ corresponds to higher concentration in W1, W4, W7 or W9 serum metabolic profiles than at baseline; ↓ to lower concentration in W1, W4, W7 or W9 serum metabolic profiles than at baseline.

After four weeks of treatment (W4), a metabolic pattern similar to the one observed at W1 discriminates treatment-related metabolic profiles from baseline in arm T+E (Figure 2D). In addition to the metabolites identified as statistically significant after one week of treatment, univariate testing highlights a significant increase in the levels of lipids (glycerol backbone of phosphoglycerides (PGLYs) and triacylglycerides (TAGs), lipoproteins mainly very low density (VLDL) and low density (LDL)) at W4, whereas acetoacetate, citrate, choline, glucose, glycerophosphocholine, myo-inositol and methanol concentrations decreased with respect to baseline. This longitudinal follow-up therefore consistently reveal that metabolic changes associated with the combined trastuzumab and everolimus treatment can be detected in sera as soon as one week after beginning the treatment (W1) and subsequently provide at W4 a stronger discrimination from baseline metabolic profiles.

### Post-treatment evolution of the metabolic profiles

**Figure 3:**
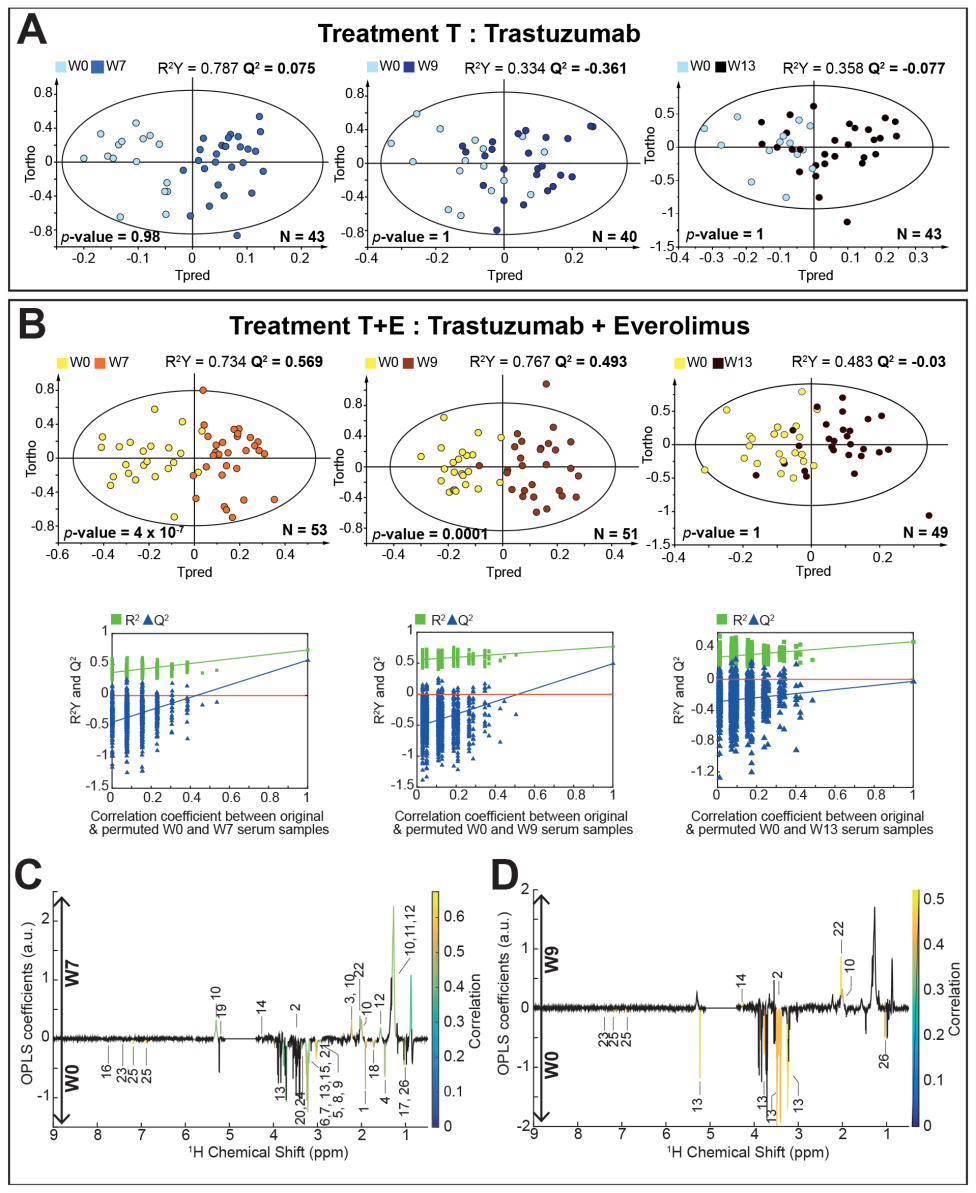
Discrimination between pre- and post-treatment serum samples **(A)** O-PLS models score plots for group T. evaluating samples at W0 vs. W7 (1+4 components, R^2^X = 0.715, R^2^Y = 0.787, Q^2^ = 0.075, CV-ANOVA *p*-value = 0.98), W0 vs. W9 (1+1 components, R^2^X = 0.531, R^2^Y = 0.334, Q^2^ = -0.361, CV-ANOVA *p*-value = 1) and W0 vs. W13 (1+1 components, R^2^X = 0.524, R^2^Y = 0.358, Q^2^ = -0.077, CV-ANOVA *p*-value = 1). **(B)** O-PLS models score plots for group T+E, discriminating samples at W0 vs. W7 (1+2 components, R^2^X = 0.625, R^2^Y = 0.734, Q^2^ = 0.569, CV-ANOVA *p*-value = 4.01 × 10^-7^), W0 vs. W9 (1+3 components, R^2^X = 0.660, R^2^Y = 0.767, Q^2^ = 0.493, CV-ANOVA *p*-value = 0.0001) and W0 vs. W13 (1+1 components, R^2^X = 0.515, R^2^Y = 0.483, Q^2^ = -0.03, CV-ANOVA *p*-value = 1). O-PLS model validations by re-sampling 1000 times the model under the null hypothesis for treatment B. **(C) and (D)** O-PLS loadings plots are represented for group T+E: W0 vs. W7 and W0 vs. W9, respectively. Statistically significant individual signals correspond to the colored spectral regions after Benjamini-Hochberg multiple testing correction. Highlighted candidate markers are: 1) Acetate, 2) Acetoacetate, 3) Acetone, 4) Alanine, 5) Albumin Lysyl, 6) Betaine, 7) Choline, 8) Creatine, 9) Creatinine, 10) Fatty acids, 11) Fatty acids (mainly LDL), 12) Fatty acids (mainly VLDL), 13) Glucose, 14) Glycerol backbone of PGLYs and TAGs, 15) Glycerophosphocholine, 16) Histidine, 17) Isoleucine, 18) Lysine, 19) Mannose, 20) Methanol, 21) Myo-inositol, 22) N-acetylglycoprotein (NAC1), 23) Phenylalanine, 24) Proline, 25) Tyrosine, 26) Valine.

Metabolomic profiles of pre- and post-treatment serum samples were compared to explore the metabolism recovery after the end of the drug intervention. As concerns treatment T, no significant discrimination was found between pre- and post-treatment serum metabolic profiles, as evaluated at W7, W9 or W13 (Figure 3A). For arm T+E, a clear discrimination of the serum metabolic profiles is observed between W0 and W7 (R^2^X = 0.625, R^2^Y = 0.734, Q^2^ = 0.569, CV-ANOVA *p-*value = 4.01 × 10^-7^) and between W0 and W9 (R^2^X = 0.660, R^2^Y = 0.767, Q^2^ = 0.493, CV-ANOVA *p-*value = 0.0001), as illustrated in Figure 3B, while the sera of patients recover to baseline metabolic profiles within seven weeks after ending the treatment, as highlighted by the absence of discrimination between W13 and W0 samples (R^2^X = 0.515, R^2^Y = 0.483, Q^2^ = -0.03, CV-ANOVA *p-*value = 1). Metabolic fingerprints taken one or three weeks after the end of treatment T+E (W7 and W9 respectively) are very close to the pattern detected during treatment at W1 and W4. In addition to the metabolites variations identified at W1 and W4, concentrations of N-aceytylglycoprotein and mannose are significantly higher in sera at W7, while a decrease in levels of isoleucine and proline is observed with respect to baseline. We note that citrate, which showed significant variation at W4, does no longer contribute to the discrimination at W7 (Figure 3C). Three weeks after ending the treatment (Figure 3D), a smaller number of metabolites reach individual statistical significance. Overall, our results show that a residual effect of everolimus and trastuzumab combined treatment can be observed on the metabolic profiles of patients several weeks after the drug intervention (up to 3 weeks). These residual perturbations of the metabolism of the host are shown to disappear within seven weeks after the end of treatment.

### Differential impact on serum metabolic profiles between treatment arms

To further compare metabolic responses associated with T and T+E treatments, further supervised analyses were carried out between T and T+E groups at the different sampling times (Supplementary Table 2). Starting from a lack of separation at baseline, a robust discrimination between treatment T and T+E-related metabolic profiles is only observed at W4 and W7, i.e. three weeks after beginning the treatment and one week after its end (Supplementary Figure 1A and 1B). As expected, corresponding metabolic patterns are very similar to those established for longitudinal evolution within group T+E (W0 vs. W1, W4, W7 or W9) in the previous section (Figure 2). Finally, metabolic profiles do no longer distinguish between arms T and T+E at W9 and W13.

## DISCUSSION

In this investigation, an untargeted metabolomics approach was applied to evaluate the impact of targeted therapies and in particular of everolimus, inhibitor of mTORC1, on the metabolism of HER-2+ breast cancer patients.

Our results first highlighted a significant serum metabolic signature associated with the combined trastuzumab and everolimus treatment, while these metabolic changes are not detected under trastuzumab intervention alone. Secondly, we showed that post-treatment residual metabolic perturbations associated with co-administration of everolimus and trastuzumab are noticeable up to 3 weeks after ending the treatment, with a gradual return to baseline profiles.

Metabolomic investigation of peripheral blood provides a snapshot of the patients’ global physiological state that reflects metabolic composition of several tissues and organs. Our work focuses on the complex interaction between host and tumor, as well as on systemic effects of the drugs on organs such as liver, muscle and visceral fat, all well-described for their important role in the control of human energetic balance and growth. Here, the metabolic signature highlighted during and after the end of treatment seems mainly associated with effects of mTOR inhibition by everolimus treatment. The metabolic response is also consistent with everolimus pharmacokinetics, which has a biological half-life of about 30 hours, i.e. is completely eliminated from the organism in about one week after ending the treatment.

**Figure 4:**
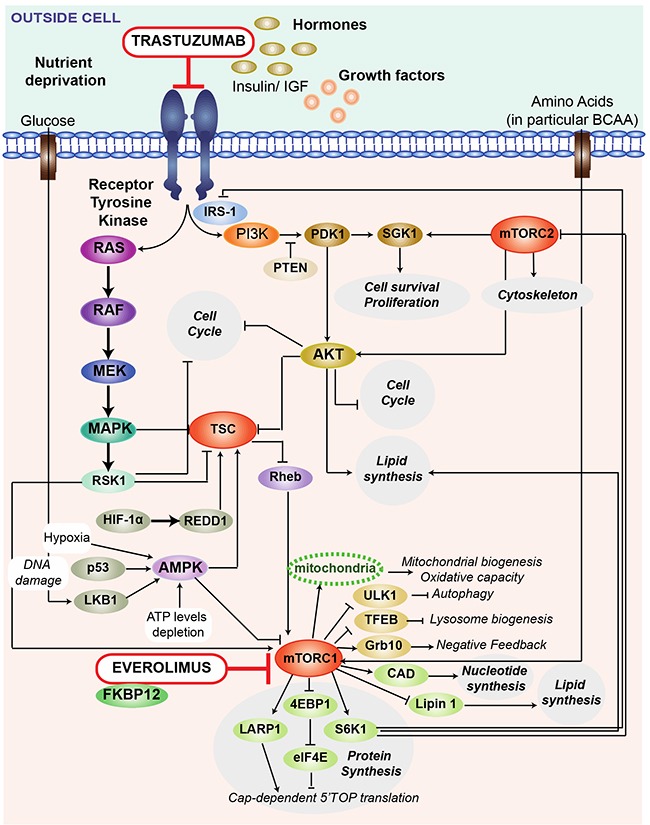
Schematic representation of the mechanisms of action for everolimus and trastuzumab Trastuzumab, a recombinant humanized monoclonal antibody, binds to the HER-2 (or c-erbB2) proto-oncogene, an extracellular domain of the human epidermal growth factor (EGF) receptor protein found on 20-30% of breast cancer cells. The binding leads to antibody-mediated killing of the HER2 positive cells. Trastuzumab inhibits the proliferation of human tumor cells that overexpress HER-2. It is a mediator of antibody dependent cellular cytotoxicity, in that the binding of the antibody to HER2 overexpressing cells leads to preferential cell death. Everolimus is a derivative of Rapamycin, and works similarly to Rapamycin as an mTOR (mammalian target of Rapamycin) inhibitor. Everolimus effect is solely on the mTORC1 protein, and not on mTORC2. Everolimus is a mTOR inhibitor that binds directly to a low-molecular-weight intracellular FKBP12 protein, thereby forming a drug complex that inhibits the activation of mTORC1. mTORC1 is a central regulator of protein synthesis, autophagy, mitochondrial function, lipogenesis, ketogenesis and glucose homeostasis in response to nutritional and environmental conditions. In a similar fashion to other mTOR inhibitors, the result of everolimus inhibition of mTOR is a reduction in cell proliferation, angiogenesis, and glucose uptake. AKT: anaplastic lymphoma kinase; CAD: CAD trifunctional protein; EGF: epidermal growth factor; FKBP12: FK506-binding protein of 12 kDa; IRS-1 : insulin receptor substrate 1; MAPK : mitogen-activating protein kinase 1; MEK : mitogen-activated protein kinase ; mTOR: mammalian target of rapamycin; LARP1: la-related protein 1; Lipin1 : lipin-1 protein; PDK1 : 3-phosphoinositide dependent protein kinase-1; PI3K: phosphatidylinositol-4,5-biphosphate-3-kinase; PTEN : phosphatase and tensin homolog; RAS : ras protein ; RAF : raf protein kinase; Rheb : ras homolog enriched in brain; RSK1 : ribosomal s6 kinase 1; S6K1 : ribosomal protein S6 beta -1; SGK1 : serum and glucorticoid-regulated kinase 1; TFEB : tanscription factor EB; TSC : tuberous sclerosis complex ; ULK1: serine/threonine protein ULK1.

The mTOR is a conserved phosphatidylinositol 3-kinase (PI3K)-like serine/threonine kinase protein that exists within two structurally and functionally distinct complexes named mTORC1 and mTORC2 [21–25]. mTORC1 promotes cell growth, proliferation and anabolism in response to nutrients (e.g. amino acids, glucose and oxygen), growth factors, cytokines and hormones (e.g. insulin/IGF-1) and cellular energy [8, 22, 25, 26]. It is found in all tissues but plays a critical role in metabolic organs (liver, muscle, and adipose tissue) to control whole body energy homeostasis leading to metabolic disorders such as obesity, type 2 diabetes and cancer. Everolimus is a rapamycin analog (rapalog) and works similarly to Rapamycin as a mTOR inhibitor. Everolimus impacts only the mTORC1 complex, and not mTORC2. However, mTORC2 can be disrupted by chronic mTOR inhibitor treatment in tissue culture as well as *in vivo* [24, 25]. Everolimus binds with high affinity to the intracellular FK506-binding protein-12 (FKBP-12) and forms a drug complex that inhibits the activation of mTORC1 complex, as illustrated in Figure 4 [27]. mTOR inhibition results in reduced cell proliferation and glucose uptake [28, 29]. Meanwhile, trastuzumab, a recombinant humanized monoclonal antibody, binds to the extracellular domain of HER-2 with high affinity, inhibiting the proliferation of human tumor cells overexpressing HER-2 (Figure 4) [30].

**Figure 5:**
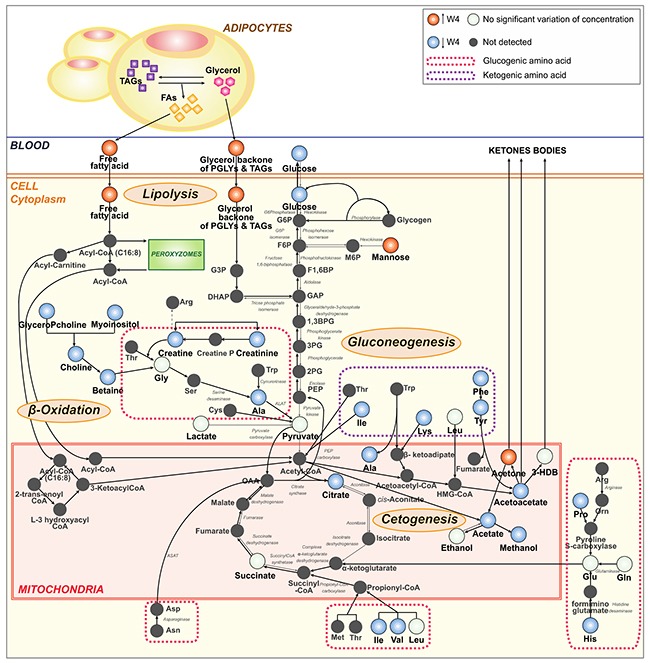
Schematic representation of the metabolic pathways linked to everolimus treatment The administration of the everolimus and trastuzumab combination, particularly of the mTOR inhibitor, in patients with HER-2+ breast cancer alters the serum host metabolome. Everolimus activates the lysis of TAGs in the adipocytes and the release of free FAs in the bloodstream. In addition, the inhibitor of mTOR promotes β-oxidationand ketogenesis. These different metabolic processes are amplified by the fasting status of patients at the time of sampling. The discussion details evidence underlying this model. The therapy empties the liver and muscle glycogen stores resulting in the decrease of glycogenolysis and gluconeogenesis. In fasting conditions, HER-2+ breast cancer patients are no longer able to maintain their blood glucose levels to reference values. The continued use of amino acids, during and after treatment, leads to a decreased gluconeogenesis. Black arrows represent the chemical reactions activated by everolimus. Red circles correspond to metabolites whose concentration are higher at W4 than at baseline. Blue circles represent metabolites with lower concentrations at W4 compared to W0, while white circles correspond to metabolites that do not vary over the intervention. 1,3BPG: 1,3-biphosphoglycerate; 3-HDB: 3-hydroxybutyrate; 2PG: 2-phosphoglycerate; 3PG: 3-phosphoglycerate; Ala: alanine; Arg: arginine; Asn: asparagine; Asp: aspartate; Creatine P: creatine phosphate; Cys: cysteine; DHAP: dihydroxyacetone phosphate; FA: fatty acid; F1,6BP: fructose-1,6-bisphosphate; F6P: fructose-6-phosphate; GAP: glyceraldehyde-3-phosphate; G3P: glycerate-3-phosphate; GlyceroPcholine: glycerophosphocholine; G6P: glucose-6-phosphate; G6Phosphatase: glucose-6-phosphatase; Gln: glutamine; Glu: glutamate; His: histidine; HMG-CoA: 3-hydroxy-3-methylglutaryl-coenzyme A; Ile: isoleucine; M6P: mannose-6-phosphate; Met: methionine; Leu: leucine; Lys: lysine; OAA: oxaloacetate; Orn: ornithine; PEP: phosphoenolpyruvate; PGLY: phosphoglyceride; Phe: phenylalanine; Pro: proline; Ser: serine; TAG: triacylglyceride; Trp: tryptophan, Tyr: tyrosine; Val: valine.

According to the literature, the mTORC1 complex plays a central role in lipids homeostasis notably by promoting lipids synthesis and storage, and by inhibiting lipids release and consumption [9, 23–25]. In addition to its role in the regulation of several transcription factors (SREBPs and Lipin1) that promote lipogenesis, mTORC1 controls adipocyte functions related to the capture of free fatty acids and storage as TAGs (Figure 5). In the presence of mTOR inhibitor, free fatty acids are not stored and remain in the bloodstream [9, 21, 23–25], which is consistent with an observed higher concentration of free fatty acids and glycerol backbone of PGLYs & TAGs in sera of patients treated with T+E. On the other hand, high levels of LDL and VLDL lipoproteins during treatment reflect the role of mTOR in the control of lipid mobilization and transport [31, 32]. A correlation between increased levels of circulating TAGs and increased levels of VLDL lipoproteins was already observed in the presence of mTOR inhibitor by Aggarwal et *al* [33].

Furthermore, mTORC1 is known to inhibit β-oxidation and ketogenesis in the liver, adipose and perhaps muscle, while instead promoting the use and storage of glucose in these tissues [21, 25]. mTOR inhibition by rapalogs highly activates β-oxidation, i.e. lipolysis, which induces the release of Acetyl-CoA that can either enter the TCA (tricarboxylic acid) cycle, or ketogenesis when the TCA cycle is stopped (e.g. in fasting conditions) [34, 35]. Ketogenesis activation by mTORC1 inhibition induces the release of ketones bodies, here acetone and acetoacetate, in the serum metabolic profiles for patients treated with T+E (Figure 5). The high production of ketone bodies during and after treatment explains the lower amounts of ketogenic amino acids (isoleucine, lysine, phenylalanine, tyrosine) as compared to baseline. Altogether, these results suggest that mTOR inhibition leads to a systemic catabolic response mimicking fasting condition. We can expect that a similar response taking place in the tumor could have therapeutic benefits by reducing cancer cell anabolism necessary for their proliferation. The different metabolic perturbations described above are likely accentuated by the fact that serum samples were collected in fasting conditions. Several studies suggest that mTORC1 signaling is respectively activated and inhibited by feeding and fasting [24, 25]. Upon fasting, stored lipids in adipose cells are released in the form of fatty acids in the bloodstream.

Meanwhile, an impaired concentration of glucose is observed for patients treated with everolimus as compared to baseline. Glucose intolerance and hyperglycemia are common side effects of mTOR inhibitors used to treat cancer, due to their critical role in glucose homeostasis [7, 36, 37]. In our study, 23.4% of the patients treated with everolimus were hyperglycemic (Supplementary Table 3), a condition likely created by the fasting-mimicking action of rapalogs known to activate liver gluconeogenesis and glycogen breakdown [22, 27]. Yet, our observations also show reduced levels of glucose for patients under everolimus treatment, with a statistically significant variation only observed at W9. Our hypothesis here is that the observed metabolic response is again largely influenced by the fasting status of the patients at the time of blood collection. Glycogenolysis is amplified by the needs, upon fasting, to maintain glucoses homeostasis in the blood and meet energy consumption in the organism. However, glycogen reserves being limited, other substrates such as gluconeogenic amino acids are progressively recruited to synthesize glucose through gluconeogenesis. After several weeks of treatment, depletion of both glycogen and gluconeogenic substrates reserves prevent patients from maintaining their blood glucose concentrations to reference levels under fasting conditions (Figure 5 and Table 2).

As the RADHER clinical trial focused on comparing the everolimus and trastuzumab combination to trastuzumab administration alone, its design did not include a subgroup of patients treated with everolimus alone. As a consequence, while available evidence correlate the observed metabolic perturbations with mTOR inhibition, we note that synergetic effects between trastuzumab and everolimus treatments cannot be completely excluded. Investigation of additional controls, including treated healthy patients, or individuals with untreated HER-2+ BC could also provide complementary assessment to our findings. Yet, these types of data are unlikely accessible within a clinical setting, and would rather require *in vitro* evaluation on a model system.

The RADHER trial overall showed successfully benefits as pre-operative treatment for HER-2+ breast cancer patients in terms of response rate [38], and combination of targeted therapies in this setting was not limited by toxicity (only 28.2% of patients in arm T+E had at least one side effect, 17.9% having a high-grade toxicity). Yet, predictive metabolic signatures of treatments response or toxicity have not been highlighted in our metabolomics investigation (Supplementary Table 1), most likely due to an insufficient population sample size. To support this hypothesis, we implemented the approach developed by Blaise et *al*. [39] to extrapolate the adequate size of a cohort suitable for detection of significant metabolite variations correlated with the response status (Supplementary Figure 2). This analysis illustrates that such predictive power could potentially be obtained from cohort sizes of hundreds to thousands of patients.

In conclusion, our work describes metabolic impacts of the everolimus and trastuzumab combination treatment for patient with HER-2+ breast cancer as pre-operative treatment. This combination induces a strong and rapid modification of the patient's metabolism as compared with trastuzumab treatment alone, with residual effects detected up to several weeks after the end of the treatment. Our findings show that mTOR inhibitor is the main cause of metabolic changes in the host metabolism. The metabolic signature observed is credibly the result of a metabolic modification of the host and possibly of the tumor that may itself explain the anti-tumor effect of the treatment. Having highlighted the potential of metabolomics approaches to study metabolic changes associated with targeted therapies, the next challenge will be to refine metabolomics capacities to predict clinical response or toxicity, towards metabolic markers-driven tailor-made therapeutic care of cancer patients.

## MATERIALS AND METHODS

### RADHER trial design and serum samples collection

From July 2008 to April 2012, 82 patients with HER-2 positive early breast cancer and accessible to surgery were enrolled in the RADHER trial. The trial aimed at determining the efficiency of the everolimus and trastuzumab combination as pre-operative treatment of HER-2+ EBC, in comparison with trastuzumab treatment alone, and studying corresponding markers of prognosis. Patients were randomized (ratio 1:1) between two different groups: group T for trastuzumab (a drip every week, up to 6 therapy cures -1 loading dose 4mg/kg then 5 × 2 mg/kg/week); and group T+E for the combination of trastuzumab and everolimus (a drip of trastuzumab every week plus 2 tablets of everolimus daily for 6 weeks – 10 mg/day) [38]. The study design is described in details in Figure 1. The local ethics committee approved the research protocol. Written informed consent was obtained for each patient before enrolment. For each patient, biological and clinicopathological data were collected including age, BMI, menopausal status, collection center, hormones receptors, size tumor residue, tumor type, SBR grade, an evaluation of the response to therapy according the Sataloff Classification [40] and toxicity (NCI CTCAE).

A series of venous blood samples were collected under fasting conditions for each patient during the RADHER trial: one before the first therapy cure (W0), two during the treatment phase i.e. after respectively one (W1) and four weeks (W4) after the beginning of treatment, and three after the 5-weeks phase of treatment, i.e. two weeks (W7), four weeks (W9) and seven weeks (W13) after the last drip of trastuzumab. Blood samples were recovered in dry tubes (5 ml) and centrifuged after 30 min of sedimentation at 3,500 rpm for 10 min at 4°C. After centrifugation, the supernatant was collected and aliquoted in three cryotubes (1 ml). Cryotubes were stored at -80°C after collection.

### ^1^H NMR spectroscopy

For NMR analysis, sera were prepared according to the Bruker standard protocol. Serum samples were thawed at room temperature before use. 300 μl of each were diluted with 300 μl of a buffer solution (0.142 Na_2_HPO_4_ wt/vol, NaN_3_ 4% vol/vol, D_2_O 10% vol/vol) in a microtube. Then, samples were centrifuged for 5 min at 4°C at 12,000 g. Finally, 550 μl of supernatant was transferred into 5 mm NMR tubes. Samples were kept for less than 24h at 4°C until analysis.

All NMR experiments were carried out on a Bruker Avance III spectrometer operating at 800.14 MHz for proton, equipped with a 5 mm TXI probe, and automatic sample changer with a cooling rack at 4°C. The temperature was then regulated at 27°C (300 K) throughout the NMR experiments. Automatic 3D shimming was performed once on a test serum sample. To ensure the good reproducibility of the data over time, additional spectra for QC serum samples were recorded. Serum QC samples were obtained by aliquoting serum from one healthy blood donor provided by the Etablissement Français du Sang. In practice, two QC serum samples were introduced respectively at the beginning and the end of each samples rack corresponding to one day of NMR throughput (~40 samples/per day) to evaluate the variability between the first and the last sample of a rack, which corresponds to about 7% QC samples in the dataset. Prior to NMR data acquisition, automatic tuning and matching, frequency locking on D_2_O and 1D automatic gradient shimming were performed on each sample. Standard ^1^H 1D NMR pulse sequences, NOESY and CPMG with water presaturation, were applied for NMR data acquisition on each sample to obtain corresponding metabolic profiles. A total of 128 transient free induction decays (FID) were collected for each experiment into 48,074 points over a spectral width of 16025.64 Hz (20 ppm). For both sequences, the acquisition time was 1.49 s, with a relaxation delay of 2 s, and the 90° pulse length was automatically calibrated for each sample at around 8.9 μs at a power level of 26 W. The NOESY mixing time was set to 10 ms and the CPMG spin-echo delay to 300 μs (for a total T2 filter of 76.8 ms) allowing an efficient attenuation of the lipid NMR signals. All FIDs were multiplied by an exponential weighting function corresponding to a 0.3 Hz line broadening factor, prior Fourier transformation

All spectra were referenced to the α-glucose anomeric proton signal (δ = 5.23 ppm). ^1^H-NMR spectra were phased and corrected for baseline using Topspin 3.1 (Bruker GmbH, Rheinstetten, Germany). After importing all 1D spectra into the AMIX software (Bruker GmbH, Rheinstetten, Germany), spectra were divided into 0.001 ppm-wide buckets to obtain 8500 × 10^3^ buckets over the chemical range of 0.5-9 ppm. Residual water signal (for NOESY spectra: 4.4 to 5.10 ppm and for CPMG spectra: 4.4 to 5.10 ppm) was excluded. Raw NMR data are available upon request to the authors. Spectra were normalized to their total intensity and Pareto scaled. We note that the normalization step was needed due to a small deviation (additional dilution) to the preparation protocol for a few samples (about 15% of the cohort), for which less than 300 μl of biological material was available. Prior to statistical analysis, spectra were aligned using the module Icoshift in Matlab (The Mathworks Inc., Natick, MA) [41].

In addition, 2D NMR experiments (^1^H-^13^C HSQC, ^1^H-^1^H TOCSY and J-Resolved experiments) were recorded on a subset of samples to achieve structural assignment of the metabolic signals. Metabolite identification procedure exploited knowledge from academic spectral databases such as HMDB [42], as well as proprietary databases (Chenomx NMR Suite v. 7.1, Chenomx Inc, Edmonton, Canada; AMIX SpectraBase v. 1.1.2, Bruker GmbH, Rheinstetten, Germany). From analysis of 1D and 2D NMR data, identification of full spin systems allowed unambiguous annotation of 49 metabolites. A ^1^H NMR CPMG mean spectrum from patients with HER-2+ early breast cancer is presented in Supplementary Figure 3 and corresponding assignments are provided in Supplementary Table 4.

### Population characterization

Descriptive statistical analysis was performed to describe the two arms, using analysis of variance for mean and χ^2^ and Fisher tests for qualitative data. The significance threshold was set up to 0.05 for all tests.

### Multivariate statistical analysis

Statistical analyses were performed on the CPMG dataset. To build models for sample classification and extract group-specific metabolic signatures, unsupervised and supervised statistical multivariate methods were conducted using SIMCA-P 13 (Umetrics, Umea, Sweden). Two types of graphs are used to visualize the data: the score and the loading plots. On the score plot, each point represents a NMR spectrum (i.e. a sample) on the main principal components, while the loading plot visualize the contribution of each NMR spectral bucket (i.e. metabolic variable) to the principal components.

Principal Component Analysis (PCA) was carried out to derive the main sources of variance and eventually identify potential outliers on the 1D ^1^H NMR datasets [43]. The high stability of the NMR setup and reproducibility of the experiments was attested by the clustered set of QC samples (data not shown).

Orthogonal partial least-squares (O-PLS) discriminant analyses were performed on the X NMR dataset matrix to discriminate samples classes by considering a supplementary data matrix Y, containing information about the sampling time (W0, W2, W4, W8, etc…) or the study arm (T or T+E) [44]. The goodness-of-fit parameters R^2^ and Q^2^, which related respectively to the explained and predicted variance, evaluated the O-PLS model performance. For each O-PLS model, a model validation in MATLAB (The MathWorks Inc., Natick, NA), using homemade O-PLS routines, was performed, by resampling the model 1000 times under the null hypothesis through random permutations of the Y matrix. The decrease of goodness-of-fit R^2^ and Q^2^ parameters, when correlation between original model and random models decreased, indicated the good quality of our model. The statistical significance of the calculated model by CV-ANOVA was also assessed for each O-PLS model [45].

To derive statistically significant associations of individual metabolites, an univariate methodology, previously described [46], that couples an automatic binning procedure named statistical recoupling of variables (SRV) to subsequent ANOVA analysis and multiple testing correction of the *p*-values was used, implemented with MATLAB homemade routines.

## SUPPLEMENTARY MATERIALS FIGURES AND TABLES






