# Supplementary material for: Longitudinal serum metabolomics evaluation of trastuzumab and everolimus combination as pre-operative treatment for HER-2 positive breast cancer patients
Source: Oncotarget. 2017 Jun 28;8(48):83570–84. doi: 10.18632/oncotarget.18784 (PMC5663537; doi:10.18632/oncotarget.18784)
Supplement: Supplementary file 1 [file oncotarget-08-83570-s001.pdf]

Longitudinal serum metabolomics evaluation of trastuzumab and everolimus combination as pre-operative treatment for HER-2 positive breast cancer patients

SUPPLEMENTARY MATERIALS

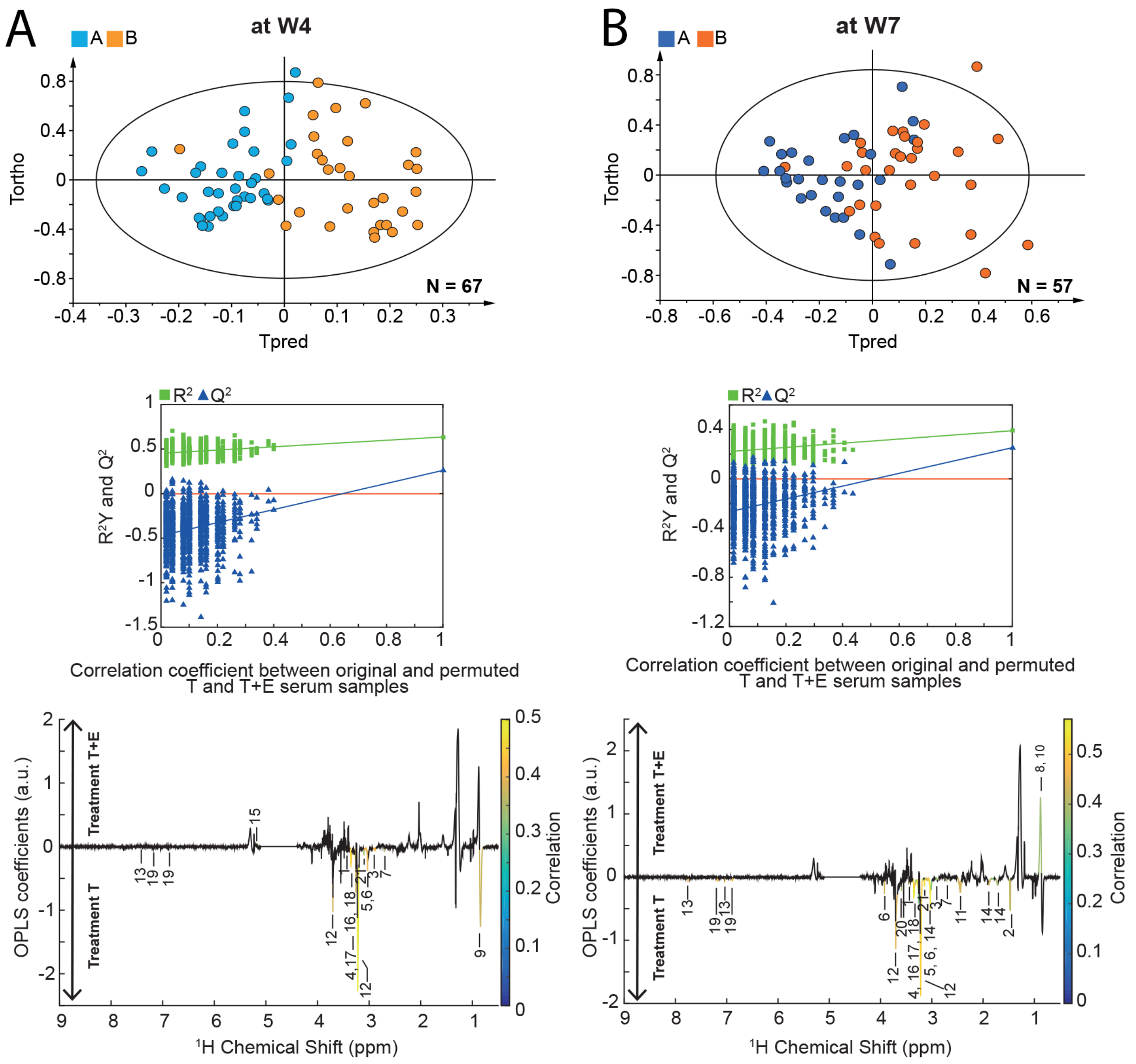

Supplementary Figure 1: Discrimination between arm T and arm T+E at W4 and W7. (A) O-PLS model at W4, discriminating T vs. T+E (1 + 3 components, $R^2X = 0.692$, $R^2Y = 0.635$, $Q^2 = 0.260$, CV-ANOVA *p*-value = 0.018). **(B)** O-PLS model at W7, discriminating T vs. T+E (1 + 1 components, $R^2X = 0.593$, $R^2Y = 0.391$, $Q^2 = 0.253$, CV-ANOVA *p*-value = 0.003). O-PLS score plots (top), models validations by re-sampling 1000 times the model under the null hypothesis (middle) and loadings plot after univariate analysis (bottom) are presented. Statistically significant signals correspond to coloured spectral regions. Highlighted candidate markers are: 1) Acetoacetate, 2) Alanine, 3) Albumin lysyl, 4) Betaine, 5) Creatine, 6) Creatinine, 7) Dimethylamine, 8) Fatty acids, 9) Fatty acids (mainly LDL), 10) Fatty acids (mainly VLDL), 11) Glutamine, 12) Glycerophosphocholine, 13) Histidine, 14) Lysine, 15) Mannose, 16) Methanol, 17) Myo-inositol, 18) Proline, 19) Tyrosine, 20) Valine and 21) Unknown 4. LDL: Very Low Density Lipoprotein; VLDL: Very Low Density Lipoprotein.

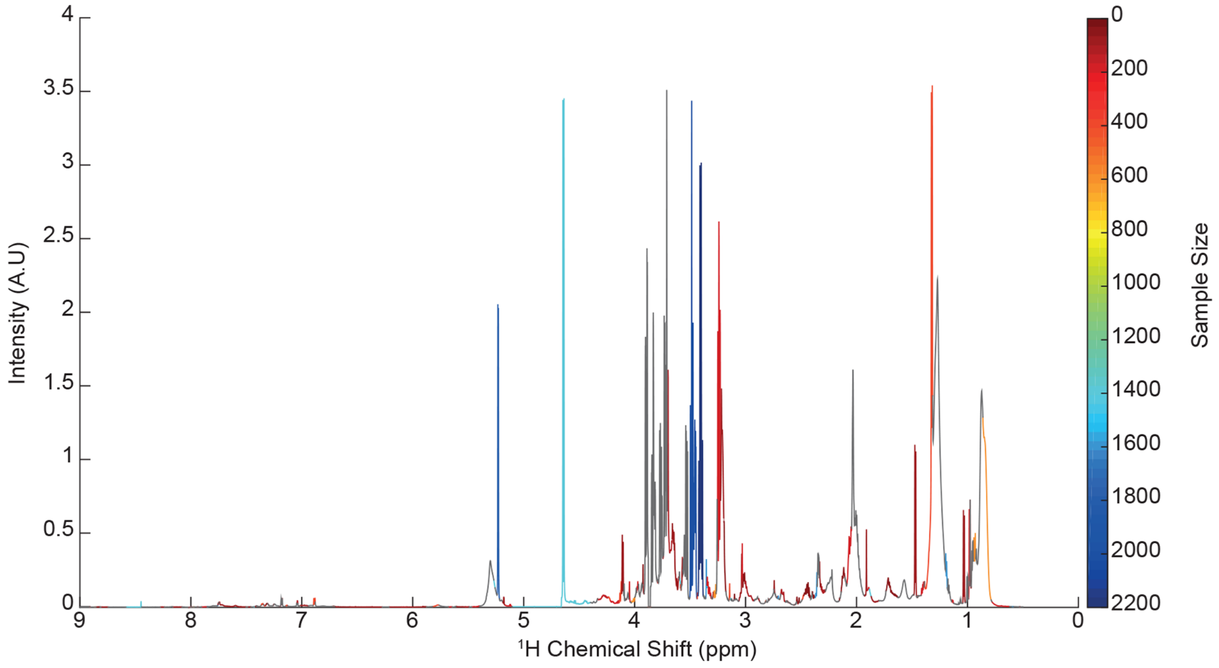

Supplementary Figure 2: Sample size determination across the NMR metabolic profile for two-classes discrimination (Complete & partial vs. no response to treatment) for treatment T+E at W0. For reference, the sample size available in the present study was N=22 (patients in arm T+E, with documented status for treatment response). Significance testing is performed with the Benjamini-Yekutieli correction. The colour node shows the necessary sample sizes for each variable to achieve a false negative rate of 0.2 (corresponding to at least one true positive with a power of 0.8). Sample size included the number of samples in the two groups. Lowest sample sizes are represented in warm colours and plotted on a typical NMR spectrum. Grey colours represent areas of the spectrum not significant or not selected by the SRV binning algorithm.

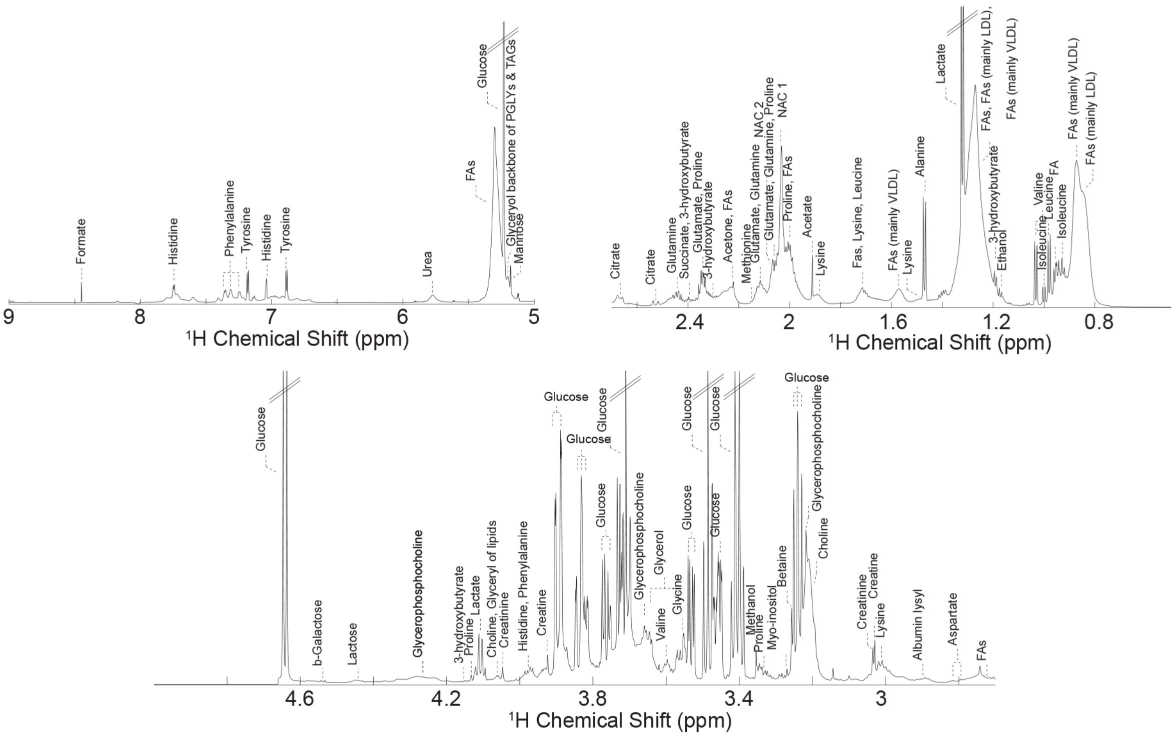

Supplementary Figure 3: Overview of the ^{1}H-NMR CPMG mean spectrum (800 MHz) for patients with HER-2 positive early breast cancer.

Supplementary Table 1: Goodness-of-fit model parameters for PLS models discriminating the histoprognosis factors of tumours, stratified by treatment and collection time of serum samples (W0, W1, W4, W7, W9 and W13).

See Supplementary file 1

Supplementary Table 2: Goodness-of-fit model parameters for O-PLS models discriminating the two treatments (T and T+E) according to the collection time of serum (W0, W1, W4, W7, W9 and W13).

Collection time	Nb of samples	Orthogonal component	R^2X	R^2Y	Q^2	*p*-value
**W0**	39	1	0.494	0.417	-0.519	1
**W1**	70	1	0.531	0.236	-0.9×10^{-3}	1
**W4**	67	3	0.692	0.635	0.26	0.018
**W7**	57	1	0.593	0.391	0.253	0.004
**W9**	52	1	0.498	0.436	0.125	0.17
**W13**	56	1	0.504	0.265	-0.12	1

Supplementary Table 3: Number of patients according to metabolic toxicity at W4 defined by NCICTC criteria for the two treatment groups.

Arm T: Trastuzumab (N = 40)					
Type toxicity	Toxicity grade [a]				
	Low grade		High grade		TOTAL
	1	2	3	4	
Hyperglycemia	4 (10%)				4 (10%)
ALAT/ASAT [b]	9 (22.5%)	1 (2.5%)			10 (25%)
Alkaline phosphatases[b]	5 (12.5%)				5 (12.5%)
Hyperlipidemia	1 (2.5%)				1(2.5%)
At least one of the above toxicities	18 (45%)				

Arm T+E: Trastuzumab + everolimus (N = 39)					
Type toxicity	Toxicity grade [a]				
	Low grade		High grade		TOTAL
	1	2	3	4	
Hyperglycemia	5 (18.2%)	1 (2.6%)	1 (2.6%)		7 (23.6%)
ALAT/ASAT[b]	6 (15.4%)	2 (5.1%)			8 (20.5%)
Alkaline phosphatases[b]	2 (5.1%)				2 (5.1%)
Hyperlipidemia	6 (15.4%)	1 (2.6%)			7 (18%)
At least one of the above toxicities	24 (61.5%)				

[a] Toxicity grade correspond to maximum intensity.
[b] The ratio of these two transaminases as well as the rate of alkaline phosphatases allow to determine whether there is liver damage; ALAT = Alanine-amino transferase and ASAT = Aspartate-amino transferase.

Supplementary Table 4: Metabolites identified from 1D and 2D NMR profiles of blood sera from patients of RADHER trial.

See Supplementary file 2
