# Supplementary material for: Longitudinal serum metabolomics evaluation of trastuzumab and everolimus combination as pre-operative treatment for HER-2 positive breast cancer patients
Source: Oncotarget. 2017 Jun 28;8(48):83570–84. doi: 10.18632/oncotarget.18784 (PMC5663537; doi:10.18632/oncotarget.18784)
Supplement: Supplementary file 2 [file oncotarget-08-83570-s002.docx]

**Supplementary Table 1: Goodness-of-fit model parameters for PLS models discriminating the histoprognosis factors of tumours, stratified by treatment and collection time of serum samples (W0, W1, W4, W7, W9 and W13).**

**PLS/PLS-DA Model**

**Treatment T (Trastuzumab)**

**Treatment T+E (Trastuzumab + Everolimus)**

**Nb of samples**

**Nb of components**

**R^2^X**

**R^2^Y**

**Q^2^**

**Nb of samples**

**Nb of components**

**R^2^X**

**R^2^Y**

**Q^2^**

**W0**

**Tumour type**

16

2

0.579

0.395

-0.194

23

2

0.484

0.456

0.064

**Hormone receptors** (HR- vs. HR+)

16

2

0.553

0.522

-0.21

23

2

0.353

0.512

-0.18

**Size tumour residue** (≤ 2 cm vs. > 2 cm)

12

2

0.629

0.769

0.053

21

2

0.5

0.641

0.135

**SBR grade** (1 & 2 vs. 3)

13

2

0.312

0.84

-0.21

20

2

0.477

0.66

0.182

**Sataloff Classification** (Complete & Partial vs. No Response)

14

2

0.627

0.746

-0.084

22

2

0.541

0.345

-0.21

**Toxicity at W4** (Grade 1 & 2 vs. 3 & 4)

/

/

/

/

/

21

2

0.467

0.658

0.181

**W1**

**Tumour type**

36

2

0.469

0.508

-0.019

34

2

0.508

0.313

-0.147

**Hormone receptors** (HR- vs. HR+)

36

2

0.483

0.388

0.058

34

2

0.542

0.301

-0.085

**Size tumour residue** (≤ 2 cm vs. > 2 cm)

27

2

0.509

0.465

-0.023

29

2

0.537

0.331

-0.21

**SBR grade** (1 & 2 vs. 3)

28

2

0.449

0.453

-0.21

29

2

0.453

0.563

0.033

**Sataloff Classification** (Complete & Partial vs. No Response)

34

2

0.465

0.466

-0.062

34

2

0.514

0.289

-0.192

**Toxicity at W4** (Grade 1 & 2 vs. 3 & 4)

/

/

/

/

/

33

2

0.236

0.409

-0.21

**W4**

**Tumour type**

36

2

0.534

0.152

-0.176

31

2

0.479

0.468

-0.21

**Hormone receptors** (HR- vs. HR+)

36

2

0.494

0.45

0.050

31

2

0.555

0.336

-0.042

**Size tumour residue** (≤ 2 cm vs. > 2 cm)

29

2

0.499

0.449

-0.012

26

2

0.376

0.362

-0.21

**SBR grade** (1 & 2 vs. 3)

28

2

0.531

0.386

-0.030

26

2

0.419

0.392

-0.15

**Sataloff Classification** (Complete & Partial vs. No Response)

36

2

0.539

0.312

-0.122

31

2

0.47

0.311

-0.21

**Toxicity at W4** (Grade 1 & 2 vs. 3 & 4)

/

/

/

/

/

31

2

0.563

0.403

-0.043

**W7**

**Tumour type**

27

2

0.532

0.339

-0.019

30

2

0.509

0.377

-0.145

**Hormone receptors** (HR- vs. HR+)

27

2

0.559

0.475

0.208

30

2

0.573

0.322

-0.147

**Size tumour residue** (≤ 2 cm vs. > 2 cm)

22

2

0.505

0.443

-0.073

26

2

0.607

0.227

-0.21

**SBR grade** (1 & 2 vs. 3)

21

2

0.325

0.642

-0.21

27

2

0.463

0.508

-0.088

**Sataloff Classification** (Complete & Partial vs. No Response)

27

2

0.537

0.400

-0.024

30

2

0.553

0.397

0.089

**Toxicity at W4** (Grade 1 & 2 vs. 3 & 4)

/

/

/

/

/

30

2

0.539

0.36

-0.143

**W9**

**Tumour type**

24

2

0.55

0.216

-0.21

28

2

0.616

0.405

0.006

**Hormone receptors** (HR- vs. HR+)

24

2

0.572

0.46

0.015

28

2

0.567

0.47

-0.07

**Size tumour residue** (≤ 2 cm vs. > 2 cm)

20

2

0.489

0.644

0.082

23

2

0.646

0.416

-0.039

**SBR grade** (1 & 2 vs. 3)

19

2

0.516

0.42

-0.21

24

2

0.56

0.712

0.168

**Sataloff Classification** (Complete & Partial vs. No Response)

24

2

0.545

0.413

-0.21

28

2

0.634

0.31

-0.093

**Toxicity at W4** (Grade 1 & 2 vs. 3 & 4)

/

/

/

/

/

28

2

0.584

0.281

-0.21

**W13**

**Tumour type**

29

2

0.567

0.327

0.127

27

2

0.54

0.38

-0.020

**Hormone receptors** (HR- vs. HR+)

29

2

0.562

0.422

-0.099

27

2

0.507

0.548

-0.052

**Size tumour residue** (≤ 2 cm vs. > 2 cm)

23

2

0.496

0.672

-0.038

22

2

0.566

0.373

-0.21

**SBR grade** (1 & 2 vs. 3)

22

2

0.55

0.637

0.274

23

2

0.506

0.788

0.623

**Sataloff Classification** (Complete & Partial vs. No Response)

29

2

0.518

0.534

-0.086

27

2

0.555

0.374

-0.618

**Toxicity at W4** (Grade 1 & 2 vs. 3 & 4)

/

/

/

/

/

27

2

0.575

0.441

-0.063
