# Supplementary material for: Longitudinal serum metabolomics evaluation of trastuzumab and everolimus combination as pre-operative treatment for HER-2 positive breast cancer patients
Source: Oncotarget. 2017 Jun 28;8(48):83570–84. doi: 10.18632/oncotarget.18784 (PMC5663537; doi:10.18632/oncotarget.18784)
Supplement: Supplementary file 3 [file oncotarget-08-83570-s003.docx]

**Supplementary Table 4: Metabolites identified from 1D and 2D NMR profiles of blood sera from patients of RADHER trial.**

**Metabolite**

**^1^H ppm**

**^13^C ppm**

**Multiplicity**

**Group**

**Observed**

**3-hydroxybutyrate**

1.19

d

ɣ-CH_3_

CPMG, JRes, TOCSY

2.30

dd

half α-CH_2_

CPMG, TOCSY

2.39

dd

half α-CH_2_

CPMG, TOCSY

4.15

m

β-CH

CPMG

**Acetate**

1.91

25.9

s

CH_3_

CPMG, JRes, TOCSY, HSQC

**Acetone**

2.22

s

CH_2_CO

CPMG, TOCSY

**Alanine**

1.47

18.8

d

CH_3_

CPMG, JRes, TOCSY, HSQC

3.77

53.5

q

α-CH

CPMG, JRes, TOCSY, HSQC

**Albumin lysyl**

2.88

42.0

t

ϵ-CH_2_

TOCSY, HSQC

2.95

41.9

t

ϵ-CH_2_

TOCSY, HSQC

3.01

42.0

t

ϵ-CH_2_

TOCSY, HSQC

**Arginine**

1.64

ɣ-CH_2_

TOCSY

1.69

ɣ-CH_2_

TOCSY

1.89

β-CH_2_

TOCSY

3.23

43.2

t

δ-CH_2_

CPMG, TOCSY, HSQC

**Aspartate**

2.66

q

half β-CH_2_

CPMG

2.80

dd

half β-CH_2_

CPMG

**Betaine**

3.26

s

CH_3_

CPMG

**Cholesterol**

0.66

m

C18 (in HDL)

TOCSY

0.68

C18 (in VLDL)

TOCSY

0.83

25.2

m

C26 and C27

CPMG, HSQC

**Choline**

3.21

56.7

s

N (CH_3_)_3_

CPMG, TOCSY, HSQC

3.51

TOCSY

4.06

TOCSY

**Citrate**

2.53

d

half CH_2_

CPMG, TOCSY

2.66

d

half CH_2_

CPMG, TOCSY

**Creatine**

3.03

s

CH_3_

CPMG, JRes, TOCSY

3.92

s

CH_2_

CPMG, JRes, TOCSY

**Creatinine**

3.04

s

CH_3_

CPMG, TOCSY

4.05

s

CH_2_

CPMG, TOCSY

**Dimethylamine**

2.71

s

CH_3_

CPMG, TOCSY

**Ethanol**

1.17

21.6

t

CH_3_

CPMG, JRes, TOCSY, HSQC

3.65

q

CH_3_COH

CPMG, TOCSY

**Fatty acids (mainly LDL)**

0.84

16.5

m

*CH_3_*(CH_2_)n

CPMG, HSQC

1.27

32.2

m

(CH_2_)n

CPMG, HSQC

**Fatty acids (mainly VLDL)**

0.86

m

*CH_3_*CH_2_CH_2_C=

CPMG, TOCSY

1.57

27.4

m

*CH_2_*CH_2_CO

CPMG, TOCSY, HSQC

1.29

m

*CH_2_*CH_2_CH_2_CO

CPMG, TOCSY

**Fatty acids**

0.93

21.05

m

*CH_3_*CH_2_

CPMG, JRes, HSQC

1.24

34.4

m

CH_3_*CH_2_*(CH_2_)n

CPMG, HSQC

1.26

25.2

m

CH_3_*CH_2_*(CH_2_)n

CPMG, TOCSY, HSQC

1.26

19.2

m

CH_2_

HSQC

1.30

m

CH_2_

CPMG, TOCSY

1.68

29.2

*CH_2_*CH_2_C=C

HSQC

2.00

29.7

m

CH_2_C=C

CPMG, TOCSY, HSQC

2.22

36.3

m

CH_2_CO

CPMG, TOCSY, HSQC

2.72

28.1

m

C=CCH_2_C=C

CPMG, TOCSY, HSQC

5.26

130.6

m

CH=*CH*CH2*CH*=CH

CPMG, HSQC

5.29

132.2

m

CH=*CH*CH2*CH*=CH

CPMG, TOCSY, HSQC

**Formate**

8.45

s

CH

CPMG, JRes, TOCSY

**Fructose**

3,99

m

CPMG

4.01

dd

CPMG

**Fucose / β-Galactose**

4.54

d

CPMG

**Glucose**

3.24

76.9

t

H2

CPMG, JRes, TOCSY, HSQC

3.40

72.4

t

H4

CPMG, JRes, TOCSY, HSQC

3.41

72.4

t

H4

CPMG, JRes, TOCSY, HSQC

3.46

78.6

m

H5

CPMG, JRes, TOCSY, HSQC

3.48

78.5

t

H3

CPMG, JRes, TOCSY, HSQC

3.53

74.3

q

H2

CPMG, JRes, TOCSY, HSQC

3.71

75.6

t

H3

CPMG, JRes, TOCSY, HSQC

3.72

63.5

q

half CH_2_-C6

CPMG, JRes, TOCSY, HSQC

3.76

63.4

m

half CH_2_-C6

CPMG, JRes, TOCSY, HSQC

3.82

74.2

ddd

H5

CPMG, JRes, TOCSY, HSQC

3.84

63.4

m

half CH_2_-C6

CPMG, JRes, TOCSY, HSQC

3.89

63.5

dd

half CH_2_-C6

CPMG, JRes, TOCSY, HSQC

4.64

98.7

d

H1

CPMG, JRes, TOCSY, HSQC

5.23

94.9

d

H1

CPMG, JRes, TOCSY, HSQC

**Glutamate**

2.04

m

half β-CH_2_

CPMG, TOCSY

2.12

m

half β-CH_2_

CPMG, TOCSY

2.34

33.9

m

half ɣ-CH_2_

CPMG, TOCSY, HSQC

2.36

m

half ɣ-CH_2_

CPMG

3.74

m

CPMG, TOCSY

**Glutamine**

2.08

TOCSY

2.09

TOCSY

2.11

29.7

m

half β-CH_2_

CPMG, JRes, TOCSY, HSQC

2.44

33.9

m

half ɣ-CH_2_

CPMG, TOCSY, HSQC

2.46

57.4

m

CPMG, HSQC

3.74

TOCSY

**Glycerol**

3.56

65.8

q

half CH_2_

CPMG, JRes, TOCSY, HSQC

3.65

65.6

q

half CH_2_

CPMG, JRes, TOCSY, HSQC

3.87

74.6

m

C_2_-H

CPMG, HSQC

**Glycerophosphocholine**

3.22

s

CPMG

3.66

68.7

m

NCH_2_

CPMG, JRes, TOCSY, HSQC

4.29

62.2

m

OCH_2_

CPMG, TOCSY, HSQC

**Glycerol backbone**

4.06

CPMG

**of PGLYs^*^ and TAGs^**^**

4.22

CHOCOR

CPMG, JRes, TOCSY

5.20

CPMG

**Glycine**

3.55

44.3

s

CH_2_

CPMG, JRes, TOCSY, HSQC

**Histidine**

3.09

dd

CPMG

3.98

dd

CPMG

7.04

s

H4

CPMG, TOCSY

7.75

s

H2

CPMG, TOCSY

**Isoleucine**

0.93

t

δ-CH_3_

CPMG, JRes, TOCSY

1.00

d

β-CH_3_

CPMG, JRes, TOCSY

1.24

half ɣ-CH_2_

TOCSY

1.46

half ɣ-CH_2_

TOCSY

1.96

TOCSY

3.65

TOCSY

**Lactate**

1.32

22.7

d

CH_3_

CPMG, JRes, TOCSY, HSQC

4.11

71.2

q

CH

CPMG, JRes, TOCSY, HSQC

**Lactose**

3.55

TOCSY

3.66

TOCSY

3.97

TOCSY

4.45

TOCSY

**Leucine**

0.95

d

δ-CH_3_

CPMG, JRes, TOCSY

0.96

d

δ-CH_3_

CPMG, JRes, TOCSY

1.66

m

CPMG

1.70

42.7

m

CPMG, TOCSY, HSQC

1.73

m

CPMG

3.71

α-CH

TOCSY

**Lysine**

1.43

m

ɣ-CH_2_

CPMG, TOCSY

1.49

m

ɣ-CH_2_

CPMG, TOCSY

1.72

m

δ-CH_2_

CPMG, TOCSY

1.88

m

β-CH_2_

CPMG, TOCSY

1.91

m

β-CH_2_

CPMG

3.02

t

CPMG, TOCSY

3.74

t

CPMG, TOCSY

**Mannose**

4.89

d

CPMG

5.18

d

CPMG

**Methanol**

3.35

s

CH_3_OH

CPMG, JRes, TOCSY

**Methionine**

2.15

s

CPMG

**Myo-inositol**

3.27

t

CPMG

**NAC^***^ 1**

2.04

24.7

s

NHCOCH_3_

CPMG, JRes, TOCSY, HSQC

**NAC^***^ 2**

2.07

25

s

NHCOCH_3_

TOCSY, HSQC

**Phenylalanine**

3.26

half β-CH_2_

TOCSY

3.97

α-CH

TOCSY

7.31

d

H2, H6

CPMG, TOCSY

7.35

m

H4

CPMG, TOCSY

7.40

m

H3, H5

CPMG, TOCSY

**Proline**

1.98

m

ɣ-CH_2_

CPMG

2.01

m

ɣ-CH_2_

CPMG, TOCSY

2.05

m

half β-CH_2_

CPMG, JRes, TOCSY

2.34

m

half β-CH_2_

CPMG, JRes, TOCSY

3.33

m

half δ-CH_2_

CPMG, JRes

4.12

m

α-CH

CPMG, JRes, TOCSY

**Succinate**

2.39

s

CPMG, JRes, TOCSY

**Threonine**

1.31

d

ɣ-CH_3_

CPMG, JRes, TOCSY

3.55

d

α-CH

CPMG, TOCSY

4.23

m

β-CH

CPMG, TOCSY

**Trehalose**

3.40

74.3

HSQC

**Tyrosine**

6.88

d

CPMG, TOCSY

7.18

d

H2, H6

CPMG, TOCSY

**Valine**

0.98

19.4

d

CH_3_

CPMG, JRes, TOCSY, HSQC

1.03

20.6

d

CH3

CPMG, JRes, TOCSY, HSQC

2.26

m

β-CH

CPMG, TOCSY

3.60

63.4

d

α-CH

CPMG, JRes, TOCSY, HSQC

**Urea**

5.77

br. s

NH_2_*C*=ONH_2_

CPMG, TOCSY

**Unknown 1**

1.39

d

CPMG, JRes, TOCSY

**Unknown 2**

1.40

d

CPMG, JRes, TOCSY

**Unknown 3**

2.74

s

CPMG, JRes, TOCSY

**Unknown 4**

3.11

s

CPMG, TOCSY

**Unknown 5**

3.14

s

CPMG, JRes, TOCSY

**Unknown 6**

7.24

d

CPMG, TOCSY

**Xylose**

3.41

78.6

HSQC

PGLYs*: Phosphoglycerides

TAGs**: Triacylglycerides

NAC***: N-acetyl-glycoprotein
